# Blood Glucose and Hemoglobin A1c Trends Pre‐, During, and Post‐COVID‐19 Pandemic in an Urban Population

**DOI:** 10.1111/1753-0407.70190

**Published:** 2026-02-10

**Authors:** Wayne Shih, Roham Hadidchi, Ekram Ali, Sonya Henry, Tim Q. Duong

**Affiliations:** ^1^ Department of Radiology Albert Einstein College of Medicine and Montefiore Health System Bronx New York USA

To the Editor,

Blood glucose level (BGL) and hemoglobin A1c are key indicators of metabolic health and predictors of diabetes‐related outcomes [1, 2, 3]. Even modest dysregulation increases morbidity and mortality, and disadvantaged urban communities such as the Bronx experience disproportionate metabolic disease burden [4, 5]. The COVID‐19 pandemic abruptly disrupted healthcare access, daily routines, and chronic disease management [6, 7]. Although early reports described short‐term increases in BGL and A1c during the initial surge [8, 9, 10, 11, 12], long‐term trajectories and whether the pandemic widened existing disparities remain unclear.

This retrospective cohort study was approved by the Einstein–Montefiore Institutional Review Board (protocol #2021‐13658). Electronic health records (EHR) from the Montefiore Health System (January 1, 2016–August 17, 2024) were analyzed as previously described [13, 14, 15, 16, 17, 18, 19, 20, 21, 22, 23, 24, 25, 26, 27].

Two cohorts were defined based on the presence or absence of type 2 diabetes mellitus (T2DM) as of January 1, 2019. All included patients had at least one recorded encounter before that date. BGL and A1c values were identified using relevant Logical Observation Identifiers Names and Codes; diagnoses used Systematic Nomenclature of Medicine Clinical Terms (see Tables S1 and S2). Monthly averages were computed from January 2019 through July 2024. Analyses were stratified by age, sex, race, ethnicity, insurance coverage, ZIP‐code income tertile, and presence of at least one unmet social need.

Patients with T2DM at baseline were older, had lower income, more unmet social needs, and higher comorbidity burden (Table S3). In both the T2DM and non‐T2DM cohorts, inpatient and outpatient testing volume declined sharply at the pandemic onset, particularly outpatient testing. Inpatient BGL and A1c values were consistently higher than outpatient values, and patients with T2DM had higher levels than those without (Figure S1).

Both BGL and A1c showed a transient spike in April 2020, returning to baseline shortly thereafter. Long‐term levels remained broadly stable, with small downward post‐pandemic offsets (Figure 1 and Table S4). Stratified analyses demonstrated similar patterns across demographic and socioeconomic groups. Males had higher glycemic values than females. Among those with T2DM, younger patients had higher BGL and A1c; among those without T2DM, older patients had higher levels. Hispanics, Blacks, and Asians had slightly higher values than Whites. Glycemic markers did not differ across ZIP‐code income tertiles, but individuals with unmet social needs had consistently higher levels. Among those with T2DM, Medicaid enrollees had the highest values; among those without T2DM, Medicare enrollees had the highest. Aside from the April 2020 spike, trends across all groups remained stable (Figures S2–S7).

**FIGURE 1:**
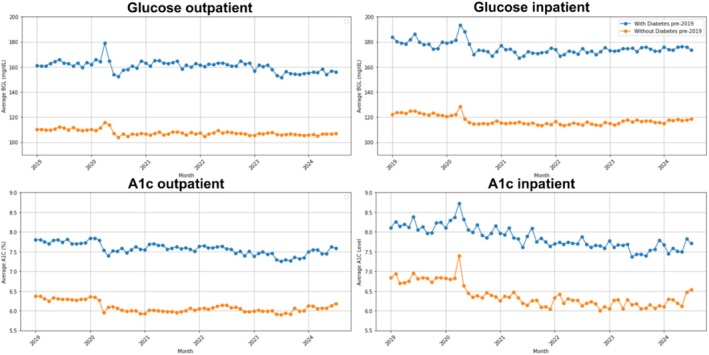
Average blood glucose level (BGL, top) and A1c (bottom) among those with (blue) and without (orange) prior type‐2 diabetes diagnosis as of January 1, 2019 for all outpatient (left) and inpatient (right) patient visits. BGL measurements are shown as mg/dL. A1c measurements are shown as %, percentages out of 100.

The early‐pandemic rise in glycemic indices likely reflects acute illness, stress hyperglycemia, cytokine‐mediated metabolic disruption, and abrupt interruptions in routine care [7, 8, 9, 10, 28, 29, 30]. Rapid normalization suggests adaptive recovery through restored access and telemedicine. Findings align with prior studies [6, 11, 12] showing transient dysregulation without sustained worsening. Our analysis expands on prior work through a long follow‐up period, a large diverse urban cohort, and detailed demographic and socioeconomic stratification. Early pandemic dysregulation was temporary, whereas longstanding disparities remained the primary drivers of glycemic differences over time.

## Author Contributions

All named authors meet the International Committee of Medical Journal Editors (ICMJE) criteria for authorship for this article and had full access to all the data in this study and take full responsibility for the integrity of the data and accuracy of the data analysis. Conceptualization: Tim Q. Duong, Wayne Shih, and Roham Hadidchi. Data analysis: Roham Hadidchi and Wayne Shih. Writing – original draft and editing: Ekram Ali, Roham Hadidchi, Wayne Shih, Tim Q. Duong, and Sonya Henry. Data curation: Sonya Henry. Supervision: Tim Q. Duong.

## Funding

The authors have nothing to report.

## Disclosure

The authors have nothing to report.

## Conflicts of Interest

The authors declare no conflicts of interest.

## Supporting information


**Data S1:** jdb70190‐sup‐0001‐Supinfo.docx.

## Data Availability

The data that support the findings of this study are available on request from the corresponding author. The data are not publicly available due to privacy or ethical restrictions.
