# Supplementary material for: Blood Glucose and Hemoglobin A1c Trends Pre‐, During, and Post‐COVID‐19 Pandemic in an Urban Population
Source: J Diabetes. 2026 Feb 10;18(2):e70190. doi: 10.1111/1753-0407.70190 (PMC12887577; doi:10.1111/1753-0407.70190)

**Supplementary Table 1:** Systematic Nomenclature of Medicine Clinical Terms (SNOMED CT) codes used for Type-2 Diabetes Mellitus

| **SNOMED Code** | **Concept Name** |
| --- | --- |
| 201826 | Type 2 diabetes mellitus |
| 4193704 | Type 2 diabetes mellitus without complication |
| 45757363 | Hypoglycemia due to type 2 diabetes mellitus |
| 201820 | Diabetes mellitus |
| 4008576 | Diabetes mellitus without complication |
| 4029423 | Hypoglycemia due to diabetes mellitus |

**Supplementary Table 2:** Logical Observation Identifiers Names and Codes (LOINC) codes for A1c and blood glucose level measurements.

| **LOINC Code** | **Concept Name** |
| --- | --- |
| 3004410 | Hemoglobin A1c/Hemoglobin.total in Blood |
| 3000483 | Glucose [Mass/volume] in Blood |
| 3004501 | Glucose [Mass/volume] in Serum or Plasma |
| 3033408 | Glucose [Mass/volume] in Venous blood |

**Supplementary Table 3.** Characteristics of patients with and without ICD-10-diagnosed type-2 diabetes prior to January 1, 2019. The χ² test was employed for group comparisons of categorical variables. For continuous variables, the independent t-test was applied. Data visualization was conducted using Matplotlib. Statistical significance was defined at a *p*-value threshold of less than 0.05. SD, standard deviation. COPD, chronic obstructive pulmonary disease.

|  | **Type-2 Diabetes by 2019 (n=41609)** | **No Type-2 Diabetes by 2019 (n=333490)** | ***p*-value** |
| --- | --- | --- | --- |
| Age at Index Date (Years), mean ± SD | 61.73 ± 14.28 | 43.58 ± 22.25 | **<0.005** |
| Female, n (%) | 25995 (62.47%) | 207526 (62.23%) | 0.33 |
| **Race and Ethnicity, n (%)** |  |  |  |
| Non-Hispanic White | 2821 (6.78%) | 33103 (9.93%) | **<0.005** |
| Black | 15371 (36.94%) | 110580 (33.16%) | **<0.005** |
| Asian | 1991 (4.79%) | 11410 (3.42%) | **<0.005** |
| Other Race | 21426 (51.49%) | 178397 (53.49%) | **<0.005** |
| Hispanic | 18432 (44.30%) | 142459 (42.72%) | **<0.005** |
| **Insurance, n (%)** |  |  |  |
| Medicaid | 12954 (31.13%) | 143938 (43.16%) | **<0.005** |
| Medicare | 14254 (34.26%) | 50703 (15.20%) | **<0.005** |
| Private | 13634 (32.77%) | 127782 (38.32%) | **<0.005** |
| Uninsured | 767 (1.84%) | 11067 (3.32%) | **<0.005** |
| **Income Tertile, n (%)** |  |  |  |
| Lower Third | 18816 (45.22%) | 139947 (41.96%) | **<0.005** |
| Middle Third | 11743 (28.22%) | 90211 (27.05%) | **<0.005** |
| Upper Third | 11050 (26.56%) | 103332 (30.99%) | **<0.005** |
| **Unmet Social Needs, n (%)** |  |  |  |
| At Least One | 4664 (11.21%) | 22145 (6.64%) | **<0.005** |
| None | 14047 (33.76%) | 88217 (26.45%) | **<0.005** |
| Unknown | 22898 (55.03%) | 223128 (66.91%) | **<0.005** |
| **Comorbidities, n (%)** |  |  |  |
| Cardiovascular Disease | 10946 (26.31%) | 12282 (3.68%) | **<0.005** |
| Hypertension | 33785 (81.20%) | 50237 (15.06%) | **<0.005** |
| Type-2 Diabetes | 41609 (100.00%) | 0 (0.00%) |  |
| COPD | 1781 (4.28%) | 2696 (0.81%) | **<0.005** |
| Asthma | 8859 (21.29%) | 26140 (7.84%) | **<0.005** |
| Chronic Kidney Disease | 10431 (25.07%) | 11524 (3.46%) | **<0.005** |
| Liver Disease | 3599 (8.65%) | 6722 (2.02%) | **<0.005** |
| **Incident Type-2 Diabetes Diagnosis, n (%)** | 0 (0.00%) | 23195 (6.96%) |  |

**Supplementary Figure 1:** Number of outpatient and inpatient blood glucose and A1c measurements in the pre-specified cohort from January 1, 2019 to July 31, 2024.


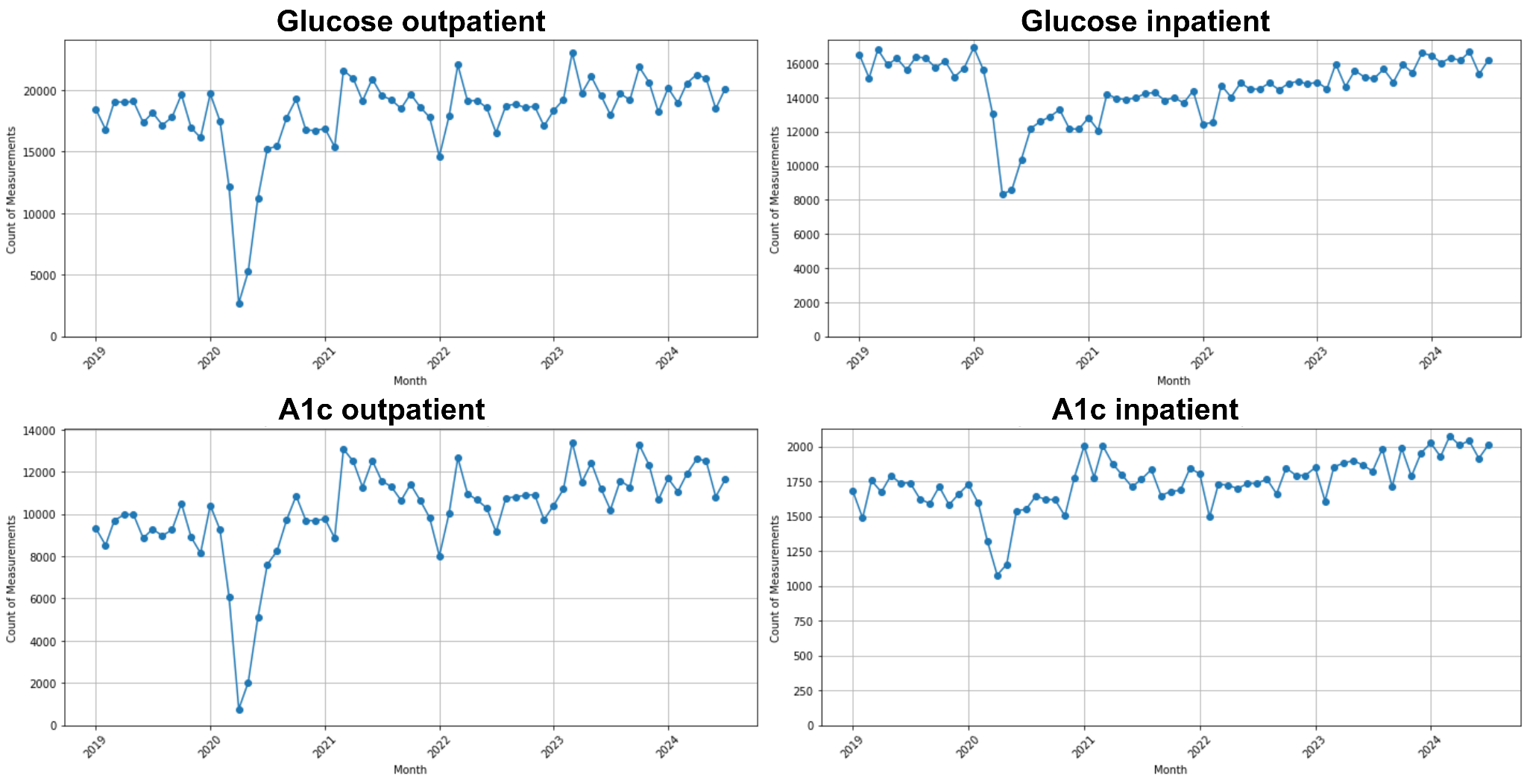


**Supplementary Table 4.** Summary of blood glucose level (BGL) and hemoglobin A1c measurements per month and averages for diabetic and non-diabetic patients in the Montefiore Health System. T2DM, type-2 diabetes.

| Year | Month | Number of Measurements | | | | Average Inpatient BGL | | Average Outpatient BGL | | Average Inpatient A1c | | Average Outpatient A1c | |
| --- | --- | --- | --- | --- | --- | --- | --- | --- | --- | --- | --- | --- | --- |
|  |  | Inpatient BGL | Outpatient BGL | Inpatient A1c | Outpatient A1c | With T2DM | Without T2DM | With T2DM | Without T2DM | With T2DM | Without T2DM | With T2DM | Without T2DM |
| 2019 | January | 16514 | 18433 | 1678 | 9316 | 183.76 | 122.27 | 161.07 | 110.21 | 8.11 | 6.83 | 7.80 | 6.37 |
| 2019 | February | 15144 | 16805 | 1486 | 8524 | 180.21 | 123.66 | 160.56 | 110.27 | 8.26 | 6.94 | 7.81 | 6.37 |
| 2019 | March | 16809 | 19086 | 1755 | 9702 | 179.05 | 123.56 | 160.83 | 109.89 | 8.15 | 6.70 | 7.75 | 6.31 |
| 2019 | April | 15924 | 19006 | 1672 | 9966 | 178.37 | 122.79 | 162.88 | 109.67 | 8.20 | 6.72 | 7.71 | 6.25 |
| 2019 | May | 16291 | 19091 | 1790 | 9978 | 182.01 | 124.75 | 164.42 | 110.54 | 8.12 | 6.75 | 7.79 | 6.33 |
| 2019 | June | 15620 | 17366 | 1735 | 8859 | 186.06 | 125.07 | 165.89 | 112.31 | 8.39 | 6.96 | 7.80 | 6.31 |
| 2019 | July | 16382 | 18171 | 1738 | 9287 | 179.69 | 123.36 | 162.95 | 111.29 | 8.05 | 6.82 | 7.74 | 6.29 |
| 2019 | August | 16306 | 17170 | 1623 | 8973 | 177.71 | 122.54 | 162.74 | 109.97 | 8.13 | 6.84 | 7.81 | 6.30 |
| 2019 | September | 15740 | 17799 | 1590 | 9252 | 178.13 | 121.86 | 160.68 | 111.92 | 7.97 | 6.83 | 7.70 | 6.29 |
| 2019 | October | 16142 | 19636 | 1711 | 10497 | 174.38 | 123.36 | 163.28 | 109.91 | 7.98 | 6.72 | 7.71 | 6.27 |
| 2019 | November | 15215 | 16940 | 1581 | 8905 | 174.71 | 121.90 | 159.46 | 109.51 | 8.23 | 6.84 | 7.71 | 6.30 |
| 2019 | December | 15711 | 16141 | 1657 | 8149 | 179.97 | 121.29 | 163.55 | 109.62 | 8.24 | 6.84 | 7.73 | 6.30 |
| 2020 | January | 16929 | 19701 | 1728 | 10413 | 179.04 | 120.40 | 161.88 | 110.12 | 8.11 | 6.82 | 7.84 | 6.36 |
| 2020 | February | 15601 | 17489 | 1594 | 9259 | 179.97 | 121.17 | 166.04 | 109.31 | 8.29 | 6.80 | 7.84 | 6.35 |
| 2020 | March | 13046 | 12157 | 1322 | 6091 | 181.29 | 122.01 | 164.34 | 111.46 | 8.37 | 6.83 | 7.79 | 6.26 |
| 2020 | April | 8348 | 2691 | 1077 | 766 | 193.46 | 128.51 | 179.02 | 115.75 | 8.72 | 7.40 | 7.53 | 5.95 |
| 2020 | May | 8557 | 5249 | 1153 | 2023 | 188.13 | 118.46 | 164.66 | 113.93 | 8.33 | 6.64 | 7.39 | 6.09 |
| 2020 | June | 10376 | 11245 | 1538 | 5107 | 178.25 | 115.68 | 153.97 | 106.98 | 8.06 | 6.45 | 7.52 | 6.10 |
| 2020 | July | 12188 | 15247 | 1548 | 7610 | 169.98 | 114.49 | 152.25 | 103.88 | 7.99 | 6.35 | 7.51 | 6.06 |
| 2020 | August | 12591 | 15433 | 1643 | 8248 | 173.33 | 114.60 | 157.40 | 106.54 | 8.18 | 6.38 | 7.59 | 6.02 |
| 2020 | September | 12887 | 17783 | 1620 | 9737 | 173.11 | 115.12 | 158.14 | 104.73 | 7.92 | 6.33 | 7.47 | 5.99 |
| 2020 | October | 13284 | 19282 | 1616 | 10862 | 172.21 | 114.37 | 160.91 | 106.69 | 7.85 | 6.45 | 7.55 | 6.00 |
| 2020 | November | 12171 | 16766 | 1501 | 9695 | 168.61 | 115.26 | 159.11 | 106.06 | 7.97 | 6.40 | 7.63 | 6.00 |
| 2020 | December | 12138 | 16716 | 1777 | 9686 | 172.48 | 117.05 | 164.85 | 107.18 | 8.15 | 6.35 | 7.56 | 5.93 |
| 2021 | January | 12810 | 16881 | 2004 | 9768 | 177.00 | 115.39 | 163.21 | 106.61 | 7.97 | 6.26 | 7.55 | 5.93 |
| 2021 | February | 12072 | 15392 | 1777 | 8866 | 173.78 | 114.93 | 160.60 | 105.67 | 7.93 | 6.36 | 7.69 | 6.02 |
| 2021 | March | 14207 | 21602 | 2003 | 13075 | 174.11 | 115.16 | 165.21 | 107.07 | 8.10 | 6.34 | 7.71 | 6.02 |
| 2021 | April | 13934 | 20957 | 1875 | 12501 | 171.78 | 115.18 | 164.99 | 108.12 | 7.85 | 6.47 | 7.67 | 6.00 |
| 2021 | May | 13880 | 19132 | 1794 | 11277 | 167.23 | 115.99 | 163.02 | 105.71 | 7.82 | 6.34 | 7.66 | 6.00 |
| 2021 | June | 13973 | 20915 | 1709 | 12537 | 168.63 | 114.79 | 162.73 | 106.77 | 7.61 | 6.20 | 7.57 | 5.97 |
| 2021 | July | 14235 | 19571 | 1764 | 11571 | 172.35 | 114.52 | 163.57 | 108.20 | 7.89 | 6.15 | 7.59 | 5.98 |
| 2021 | August | 14280 | 19201 | 1833 | 11292 | 171.23 | 115.26 | 164.78 | 108.04 | 8.10 | 6.25 | 7.63 | 5.95 |
| 2021 | September | 13833 | 18511 | 1646 | 10642 | 170.86 | 113.78 | 158.28 | 107.58 | 7.75 | 6.27 | 7.57 | 5.98 |
| 2021 | October | 13997 | 19696 | 1677 | 11410 | 171.64 | 113.17 | 161.36 | 105.65 | 7.84 | 6.09 | 7.60 | 6.00 |
| 2021 | November | 13678 | 18626 | 1685 | 10646 | 171.98 | 115.15 | 159.89 | 107.63 | 7.75 | 6.10 | 7.56 | 6.07 |
| 2021 | December | 14396 | 17792 | 1842 | 9826 | 174.99 | 114.08 | 162.62 | 106.53 | 7.64 | 6.05 | 7.52 | 6.02 |
| 2022 | January | 12429 | 14623 | 1804 | 8018 | 173.83 | 116.47 | 161.59 | 107.33 | 7.70 | 6.33 | 7.64 | 6.05 |
| 2022 | February | 12537 | 17880 | 1494 | 10029 | 168.83 | 114.28 | 160.45 | 104.49 | 7.74 | 6.42 | 7.65 | 6.07 |
| 2022 | March | 14710 | 22056 | 1729 | 12664 | 169.81 | 113.40 | 162.37 | 106.47 | 7.69 | 6.20 | 7.60 | 6.04 |
| 2022 | April | 14019 | 19117 | 1721 | 10960 | 172.89 | 114.33 | 161.83 | 107.56 | 7.74 | 6.31 | 7.60 | 6.07 |
| 2022 | May | 14841 | 19166 | 1697 | 10671 | 172.01 | 115.17 | 163.24 | 109.48 | 7.72 | 6.27 | 7.63 | 6.12 |
| 2022 | June | 14498 | 18580 | 1734 | 10289 | 170.32 | 114.59 | 163.28 | 107.21 | 7.70 | 6.27 | 7.63 | 6.14 |
| 2022 | July | 14483 | 16543 | 1734 | 9153 | 174.75 | 113.75 | 162.11 | 108.01 | 7.88 | 6.14 | 7.57 | 6.15 |
| 2022 | August | 14855 | 18725 | 1766 | 10751 | 171.41 | 116.08 | 160.91 | 107.86 | 7.68 | 6.19 | 7.56 | 6.07 |
| 2022 | September | 14449 | 18869 | 1659 | 10813 | 172.58 | 114.65 | 160.76 | 106.80 | 7.61 | 6.23 | 7.46 | 6.09 |
| 2022 | October | 14820 | 18579 | 1845 | 10886 | 169.89 | 113.84 | 164.71 | 107.03 | 7.66 | 6.18 | 7.50 | 6.05 |
| 2022 | November | 14947 | 18689 | 1792 | 10915 | 172.12 | 113.25 | 162.30 | 106.76 | 7.65 | 6.01 | 7.40 | 5.98 |
| 2022 | December | 14784 | 17089 | 1792 | 9735 | 175.60 | 115.92 | 163.19 | 105.48 | 7.59 | 6.10 | 7.51 | 5.98 |
| 2023 | January | 14894 | 18348 | 1848 | 10413 | 172.90 | 114.90 | 156.61 | 105.60 | 7.78 | 6.06 | 7.38 | 5.99 |
| 2023 | February | 14509 | 19235 | 1608 | 11182 | 172.63 | 113.91 | 161.65 | 106.97 | 7.61 | 6.27 | 7.45 | 6.02 |
| 2023 | March | 15965 | 23045 | 1853 | 13392 | 173.28 | 114.92 | 160.27 | 106.60 | 7.67 | 6.29 | 7.49 | 5.98 |
| 2023 | April | 14636 | 19772 | 1880 | 11519 | 174.51 | 116.82 | 161.68 | 107.53 | 7.66 | 6.05 | 7.43 | 5.99 |
| 2023 | May | 15573 | 21098 | 1898 | 12427 | 174.77 | 117.83 | 157.89 | 107.74 | 7.69 | 6.28 | 7.46 | 6.00 |
| 2023 | June | 15188 | 19546 | 1865 | 11195 | 174.89 | 116.33 | 153.15 | 106.11 | 7.37 | 6.16 | 7.30 | 5.91 |
| 2023 | July | 15103 | 17992 | 1822 | 10200 | 172.47 | 117.93 | 151.67 | 105.86 | 7.44 | 6.18 | 7.26 | 5.90 |
| 2023 | August | 15661 | 19727 | 1982 | 11581 | 175.35 | 116.52 | 156.48 | 106.28 | 7.44 | 6.05 | 7.29 | 5.94 |
| 2023 | September | 14897 | 19205 | 1709 | 11273 | 175.86 | 116.99 | 154.74 | 106.59 | 7.40 | 6.07 | 7.27 | 5.92 |
| 2023 | October | 15930 | 21879 | 1986 | 13272 | 174.31 | 117.07 | 154.18 | 106.21 | 7.53 | 6.15 | 7.35 | 6.07 |
| 2023 | November | 15444 | 20648 | 1785 | 12332 | 172.61 | 115.86 | 154.13 | 105.86 | 7.56 | 6.06 | 7.32 | 5.99 |
| 2023 | December | 16599 | 18222 | 1951 | 10678 | 172.57 | 115.93 | 154.86 | 105.29 | 7.79 | 6.13 | 7.35 | 6.00 |
| 2024 | January | 16458 | 20173 | 2024 | 11688 | 175.82 | 115.01 | 155.23 | 105.91 | 7.68 | 6.10 | 7.50 | 6.13 |
| 2024 | February | 16020 | 18953 | 1926 | 11060 | 173.80 | 117.68 | 155.89 | 106.04 | 7.44 | 6.30 | 7.55 | 6.12 |
| 2024 | March | 16331 | 20570 | 2074 | 11905 | 173.51 | 117.23 | 155.48 | 104.94 | 7.59 | 6.28 | 7.55 | 6.06 |
| 2024 | April | 16166 | 21261 | 2008 | 12602 | 175.81 | 118.18 | 158.18 | 106.43 | 7.51 | 6.19 | 7.45 | 6.07 |
| 2024 | May | 16676 | 20978 | 2041 | 12531 | 176.28 | 117.53 | 154.09 | 106.72 | 7.49 | 6.12 | 7.45 | 6.07 |
| 2024 | June | 15376 | 18496 | 1914 | 10818 | 176.05 | 117.72 | 156.63 | 106.60 | 7.82 | 6.48 | 7.63 | 6.13 |
| 2024 | July | 16219 | 20119 | 2010 | 11640 | 173.29 | 118.51 | 156.07 | 107.01 | 7.71 | 6.54 | 7.58 | 6.18 |

**Supplementary Figure 2.** Average outpatient and inpatient blood glucose level (BGL) and hemoglobin A1c values stratified by sex and type-2 diabetes diagnosis as of January 1, 2019. BGL measurements are shown as mg/dL. A1c measurements are shown as %, percentages out of 100.
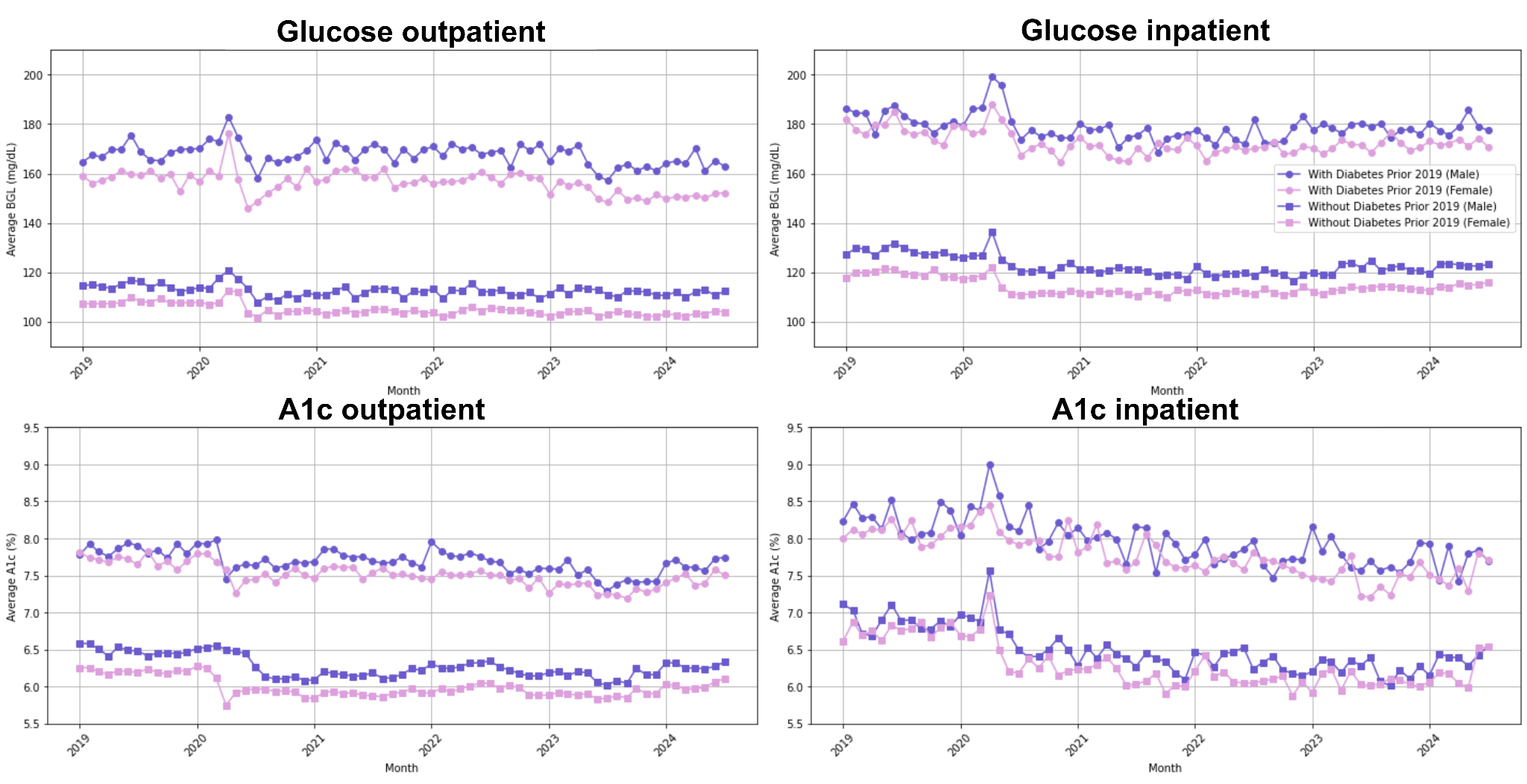


**Supplementary Figure 3.** Average outpatient and inpatient blood glucose level (BGL) and hemoglobin A1c values stratified by age (≥60, <60) and type-2 diabetes diagnosis as of January 1, 2019. BGL measurements are shown as mg/dL. A1c measurements are shown as %, percentages out of 100


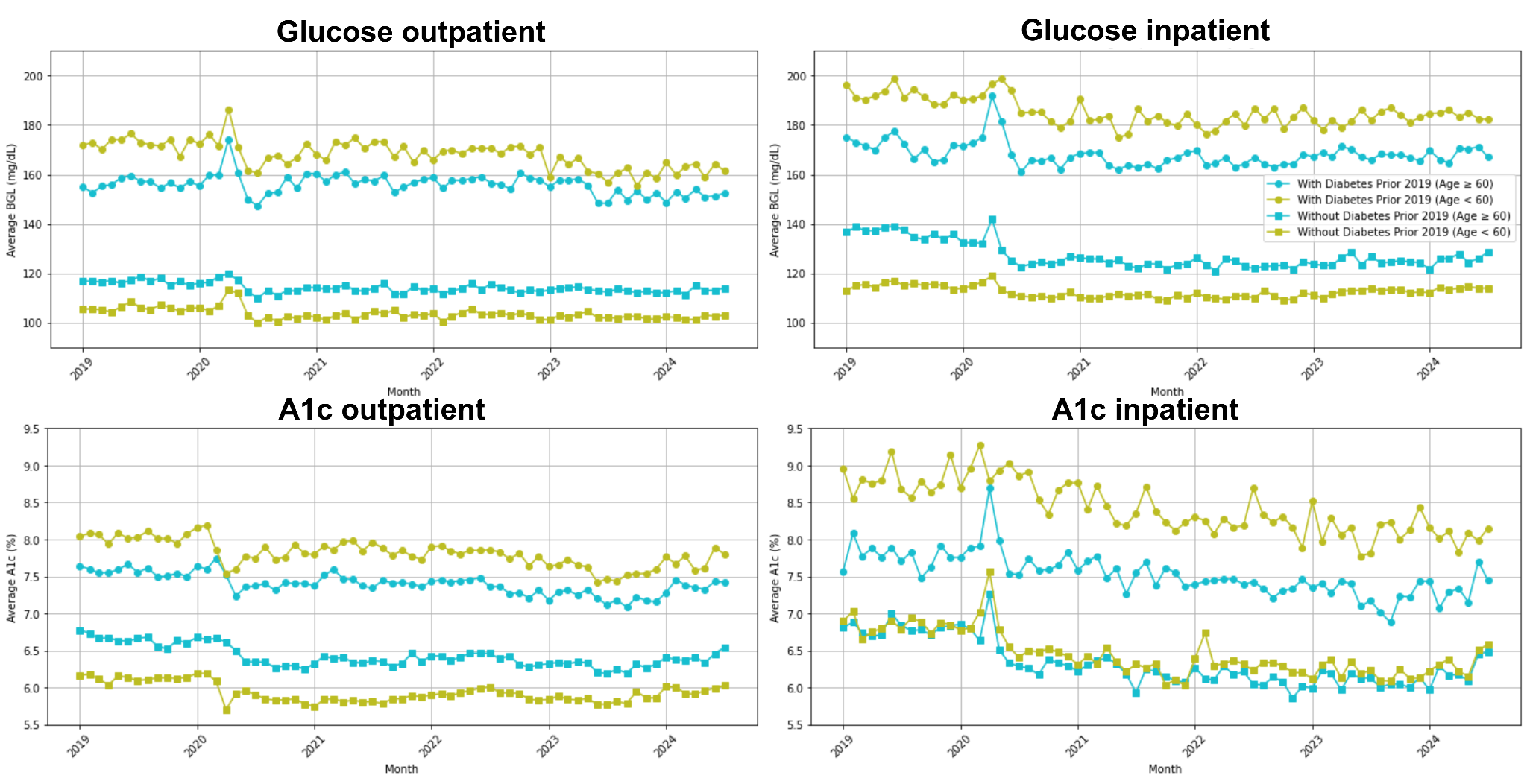


**Supplementary Figure 4.** Average outpatient and inpatient blood glucose level (BGL) and hemoglobin A1c values stratified by race (Asian, Black, White, Hispanic) and type-2 diabetes diagnosis as of January 1, 2019. BGL measurements are shown as mg/dL. A1c measurements are shown as %, percentages out of 100.


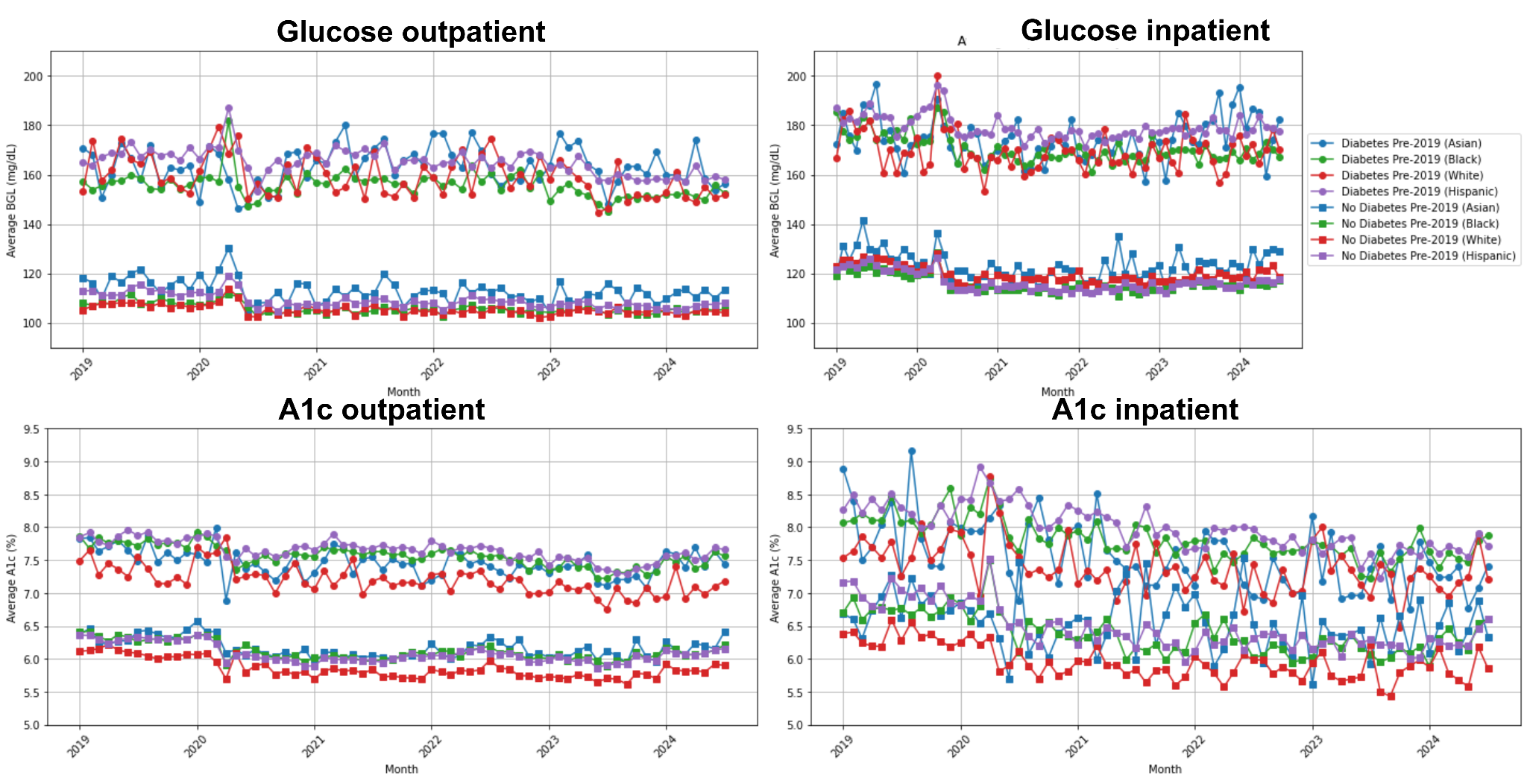


**Supplementary Figure 5.** Average outpatient and inpatient blood glucose level (BGL) and hemoglobin A1c values stratified by Zone Improvement Plan median income tertile (low third, middle third, upper third) and type-2 diabetes diagnosis as of January 1, 2019. BGL measurements are shown as mg/dL. A1c measurements are shown as %, percentages out of 100.


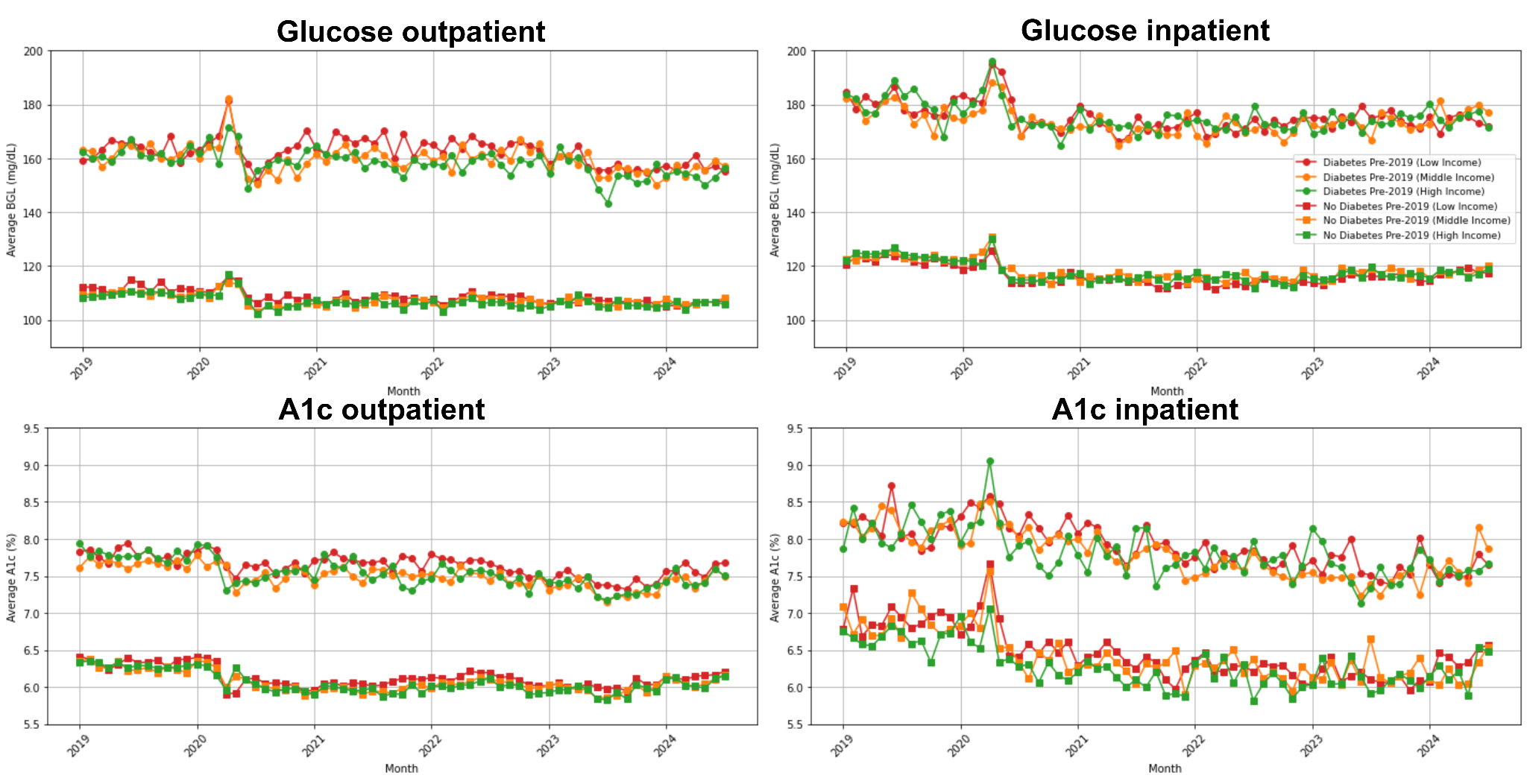


**Supplementary Figure 6.** Average outpatient and inpatient blood glucose level (BGL) and hemoglobin A1c values stratified by social needs status (met, unmet) and type-2 diabetes diagnosis as of January 1, 2019. BGL measurements are shown as mg/dL. A1c measurements are shown as %, percentages out of 100.


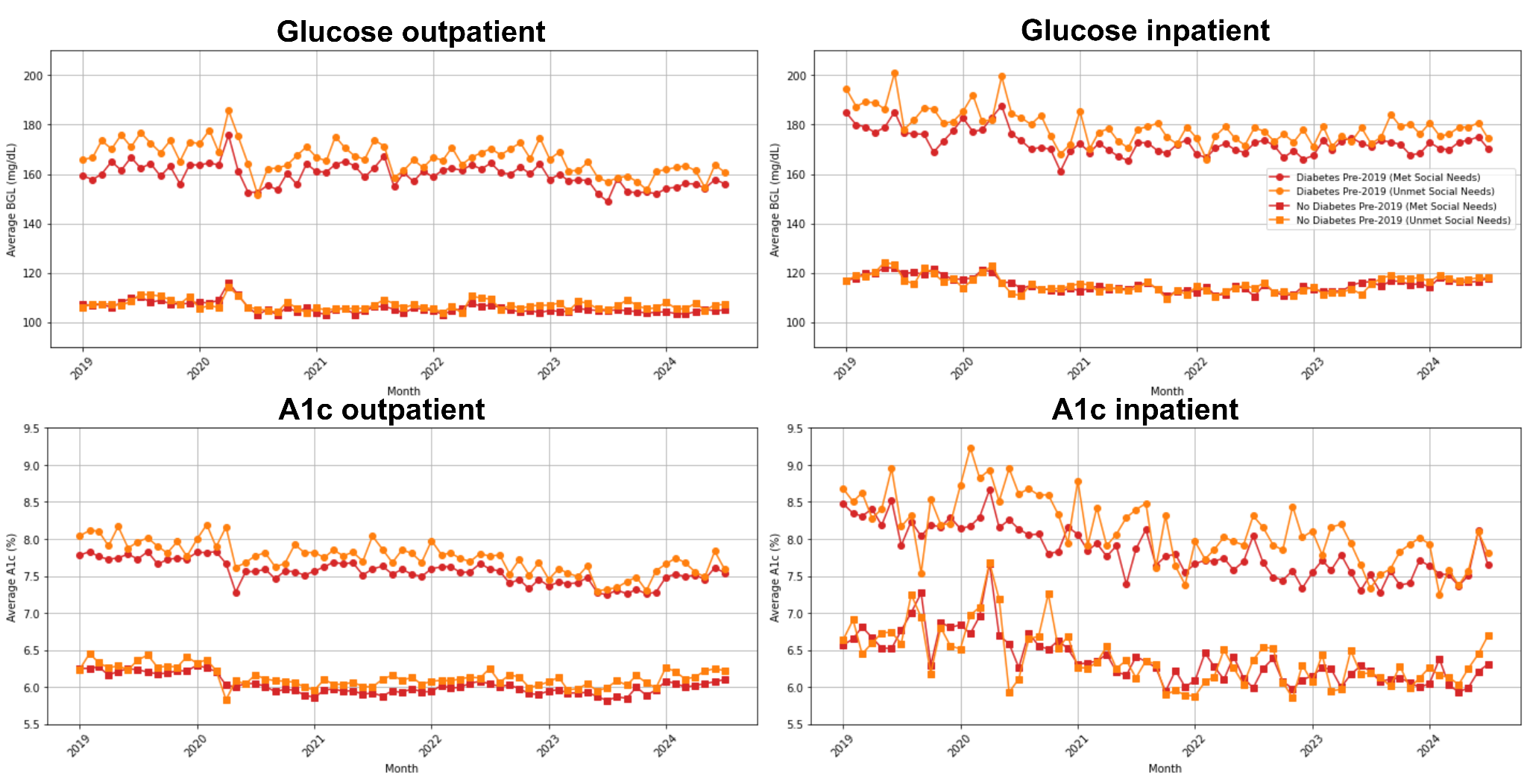


**Supplementary Figure 7.** Average outpatient and inpatient blood glucose level (BGL) and hemoglobin A1c values stratified by insurance coverage (Medicaid, Medicare, private, uninsured) and type-2 diabetes diagnosis as of January 1, 2019. BGL measurements are shown as mg/dL. A1c measurements are shown as %, percentages out of 100. The data of the uninsured group were “noisy” because of small sample size.


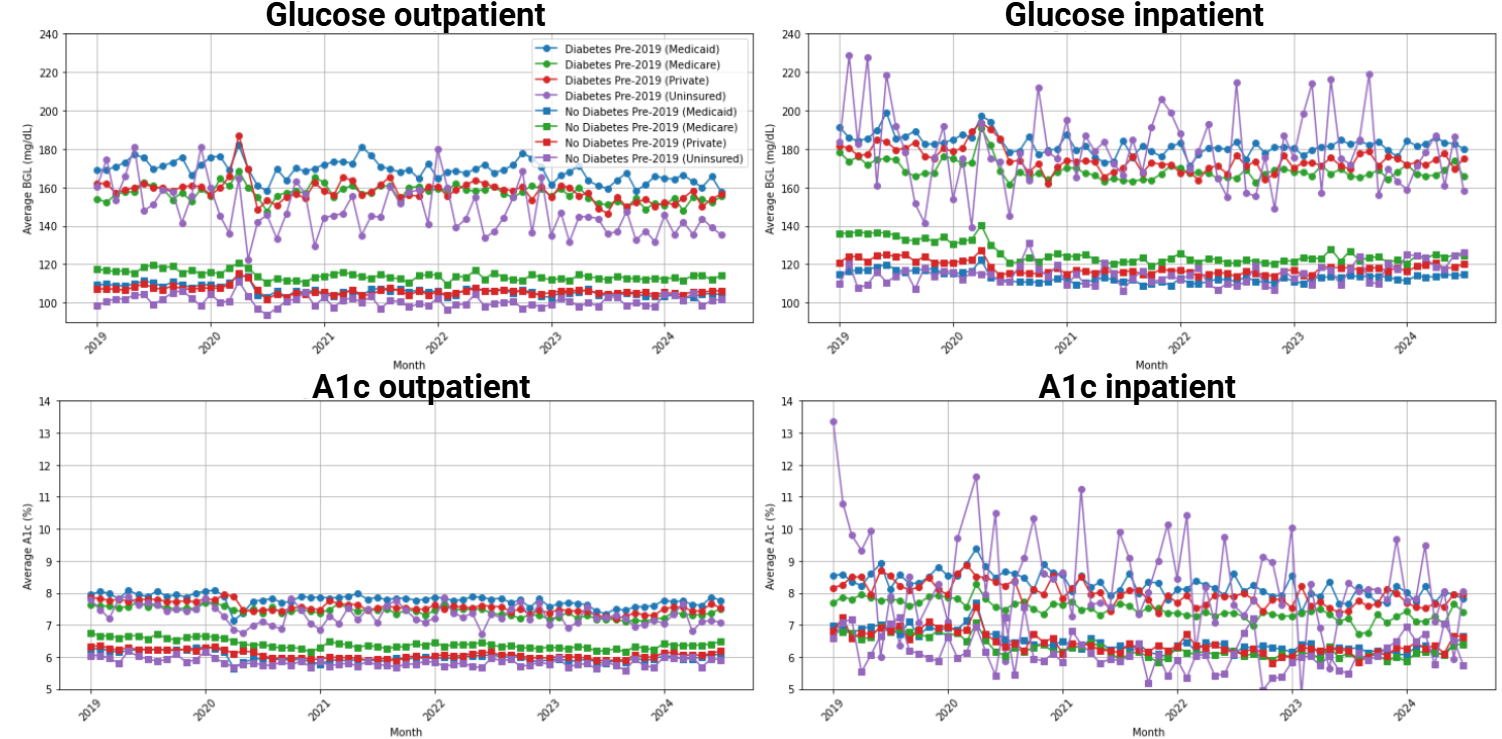

Supplement: Supplementary file 1 — Data S1: jdb70190‐sup‐0001‐Supinfo.docx. [file JDB-18-e70190-s001.docx]
